# Pediatric Non-Alcoholic Fatty Liver Disease: Nutritional Origins and Potential Molecular Mechanisms

**DOI:** 10.3390/nu12103166

**Published:** 2020-10-16

**Authors:** Ashok Mandala, Rachel C. Janssen, Sirish Palle, Kevin R. Short, Jacob E. Friedman

**Affiliations:** 1 Harold Hamm Diabetes Center, University of Oklahoma Health Sciences Center, Oklahoma City, OK 73104, USA; ashok-mandala@ouhsc.edu (A.M.); rachel-janssen@ouhsc.edu (R.C.J.); kevin-short@ouhsc.edu (K.R.S.); 2 Department of Pediatrics, Section of Gastroenterology, Hepatology & Nutrition, University of Oklahoma Health Sciences Center, Oklahoma City, OK 73104, USA; sirish-palle@ouhsc.edu; 3 Department of Pediatrics, Section of Diabetes and Endocrinology, University of Oklahoma Health Sciences Center, Oklahoma City, OK 73104, USA; 4 Department of Physiology, University of Oklahoma Health Sciences Center, Oklahoma City, OK 73104, USA

**Keywords:** pediatric NAFLD, clinical biomarkers, microRNAs, developmental programming, microbial dysbiosis, trained immunity

## Abstract

Non-alcoholic fatty liver disease (NAFLD) is the number one chronic liver disease worldwide and is estimated to affect nearly 40% of obese youth and up to 10% of the general pediatric population without any obvious signs or symptoms. Although the early stages of NAFLD are reversible with diet and lifestyle modifications, detecting such stages is hindered by a lack of non-invasive methods of risk assessment and diagnosis. This absence of non-invasive means of diagnosis is directly related to the scarcity of long-term prospective studies of pediatric NAFLD in children and adolescents. In the majority of pediatric NAFLD cases, the mechanisms driving the origin and rapid progression of NAFLD remain unknown. The progression from NAFLD to non-alcoholic steatohepatitis (NASH) in youth is associated with unique histological features and possible immune processes and metabolic pathways that may reflect different mechanisms compared with adults. Recent data suggest that circulating microRNAs (miRNAs) are important new biomarkers underlying pathways of liver injury. Several factors may contribute to pediatric NAFLD development, including high-sugar diets, in utero exposures via epigenetic alterations, changes in the neonatal microbiome, and altered immune system development and mitochondrial function. This review focuses on the unique aspects of pediatric NAFLD and how nutritional exposures impact the immune system, mitochondria, and liver/gastrointestinal metabolic health. These factors highlight the need for answers to how NAFLD develops in children and for early stage-specific interventions.

## 1. Introduction

Non-alcoholic fatty liver disease (NAFLD) is a generic term that describes a spectrum of diseases including non-alcoholic fatty liver (NAFL), non-alcoholic steatohepatitis (NASH), fibrosis, and NAFLD-cirrhosis [1]. The global epidemic of NAFLD is increasing exponentially owing to the growing prevalence of obesity and type 2 diabetes (T2D) in children and adults along with the aging population [2]. Recent estimates indicate that the global prevalence of NAFLD is 25%, with the highest prevalence in the Middle East and South America and the lowest in Africa [3]. NAFLD is the most common liver disorder and currently is the second most common cause of liver transplantation [4]. NAFLD is estimated to affect 34% of obese children aged 2–19 years and 10% of the general pediatric population [5]. Pediatric NAFLD is associated with extrahepatic complications such as early atherosclerosis and cardiac dysfunction [6,7] and abnormal renal function [8]. Recently, NAFLD has been renamed by some as metabolic (dysfunction) associated fatty liver disease (MAFLD) as the majority of patients with fatty liver have metabolic dysfunction in the form of T2D, dyslipidemia, and increased insulin resistance [9]. Pediatric NAFLD has a complex pathophysiology and is different from adult NAFLD with multiple inputs, including perinatal events. Understanding these differences may lead to new biomarkers and opportunities for novel therapeutics.

## 2. Clinical Pathophysiology

Despite advances in understanding NAFLD in adults, major gaps remain in defining pathways and mechanisms unique to NAFLD pathology in children. The pathophysiology of pediatric NAFLD is multi-factorial and includes complex interactions among hormonal, nutritional, genetic, and environmental factors [10] that may begin in utero [11]. Initially, NAFLD involves hepatic steatosis, which comprises lipid accumulation arising from excessive influx of fatty acids from endogenous fat depots, excess consumption of dietary fat, and hepatic de novo lipogenesis (DNL). NASH is characterized by inflammation, oxidative stress, mitochondrial dysfunction, and fibrosis [12].

Guidelines for diagnosing NAFLD in children were updated in 2017 by the North American Society of Pediatric Gastroenterology, Hepatology and Nutrition (NASPGHAN) [13]. That expert group emphasized that obese children should be prioritized for screening because of their higher likelihood to have NAFLD. They also recognized that an unmet challenge is to identify reliable and minimally invasive biomarkers for the disease. The only currently NAPSGHAN-recommended blood test for screening for pediatric NAFLD is alanine aminotransferase (ALT). The American Academy of Pediatrics endorsed the NASPGHAN recommendation to measure ALT beginning at ages 9–11 years for all obese children, and for overweight children with additional risk factors such as insulin resistance, diabetes, dyslipidemia, sleep apnea, central adiposity, or a family history of NAFLD [14]. NAFLD is likely present in obese children who have ALT values that are 2-fold higher than the sex-specific normal range. An advantage of ALT is that it is inexpensive, and the measurement has been standardized among laboratories. However, normal ranges reported among locations are variable, which complicates interpretation, and, more importantly, ALT values do not reliably differentiate NAFLD severity, or distinguish uncomplicated NAFLD from NASH in children or adults [13,15,16]. Several other potential circulating biomarkers have been proposed [17,18,19,20,21,22,23,24] but few have been tested in children with biopsy-proven NAFLD, or tracked over the course of treatment or disease progression [13,16,25].

Imaging tools have been used for screening, but most approaches have recognized limitations [13,24]. Standard B-mode ultrasound lacks specificity and sensitivity for steatosis and is not recommended [13]. Computed tomography and magnetic resonance (MR) modalities are better than ultrasound; however, they carry concerns about radiation (the former), expense and availability of instrumentation (the latter), and the need for sedation in some children (both) [13]. Newer imaging tools that are gaining acceptance and application include MR elastography, ultrasound-based shear wave elastography using acoustic radiation force impulse (ARFI) techniques, and ultrasound-based vibration-controlled transient elastography (FibroScan) [26,27]. MR elastography has not yet been shown to be as useful in children as it has in adults and requires an MRI machine and a specific surface coil, so it is likely to be implemented only in medical centers with specialty clinics. ARFI was shown to be useful in determining liver fibrosis in pediatric patients with chronic liver disease [28] and showed high correlation with aspartate aminotransferase (AST)/ALT ratios and detecting NAFLD in childhood obesity [29]. The FibroScan has been validated for measuring liver steatosis in adults and children and is FDA approved for clinical and research applications [24,30]. There are small, medium, and extra-large probes that can be selected to accommodate the size of the patient, which obviously spans a large potential range from younger children to adolescents and young adults. Like MR-based approaches, it is not yet widely available in pediatric clinics [31]. Thus, imaging techniques have promise but additional tools are needed for comprehensive liver health profiling.

The current gold standard for confirmation of NAFLD is histological examination of liver tissue obtained by biopsy, as it rules out other causes of liver dysfunction [13]. Histological differences have been demonstrated in pediatric versus adult NAFLD, with children being more likely to display portal inflammation and fibrosis and less ballooning and peri-sinusoidal fibrosis than adults [25]. Whether those distinctions result in different trajectories of disease progression or responses to treatment is not yet known. It should be noted that, in a separate clinical guideline for pediatric NAFLD released by the American Association for the Study of Liver Diseases in 2018, there were no recommended blood or imaging tests for screening for NAFLD in obese children because of the paucity of evidence [32]. Recommendations for clinical work up and liver biopsy, however, were similar to those made by NASPGHAN [26].

Bile acids are commonly studied as biomarkers and therapeutic targets for NAFLD. Bile acids are synthesized from cholesterol in the liver and are the major components of bile. Altered bile acid composition and metabolism have been reported during the progression of NAFLD [33]. Currently, little evidence exists linking the development of cholestasis with NAFLD/NASH. Metabolomic analysis revealed significantly increased serum levels of glycochenodeoxycholate, glycocholate, and taurocholate in patients with NAFLD compared with healthy controls [34]. Research in this field is complicated by the complexity of the liver-bile-intestinal axis and is therefore more focused on pharmacotherapies like the FXR-agonist, obeticholic acid, and peroxisome proliferator-activated receptor (PPAR) agonists such as saroglitazar rather than on bile acids as clinical biomarkers. Free fatty acids and their metabolites, which contribute to liver injury via increased oxidative stress, are typically elevated in children and adults with obesity and NAFLD [35,36,37,38]. As with bile acids, free fatty acids are mostly used as therapeutic targets rather than clinical biomarkers.

Genetic factors are associated with NAFLD susceptibility and progression. A variant in the patatin-like phospholipase domain-containing protein 3 gene (*PNPLA3*) is associated with increased liver fat, fibrosis, and risk for carcinoma, with a higher prevalence of the at-risk allele in Hispanic youth [39,40]. A variant in the glucokinase regulatory protein (*GCKR*) gene was associated with an increased rate of DNL in obese adolescents [41], and a minor allele in the transmembrane 6 superfamily 2 human gene (*TM6SF2*) was associated with higher fibrosis and NAFLD Activity Score in children [42]. Lysosomal acid lipase (LAL) deficiency is observed in two recessive genetic disorders involving increased lysosomal cholesterol ester storage. LAL activity was shown to be significantly reduced in children [43] and adults [44,45] with NAFLD, suggesting a possible role of LAL reduction in the progression of NAFLD [46]. It is not yet known whether the presence of these polymorphisms modify the response to lifestyle or pharmacological interventions designed to slow or reverse the development and progression of NAFLD. However, Van Name et al. [47] demonstrated that a small group (*n* = 17) of obese children who completed a 12-week diet with low n-6/n-3 fatty acids had favorable changes in lipids, liver fat, and insulin sensitivity and these changes were the same or slightly better in patients with the *PNPLA3* “at risk” genotype.

## 3. Role of Nutrients in Pediatric NAFLD

Animal and human evidence supports the adverse effects of high sugar intake, particularly fructose, on obesity and pediatric NAFLD risk, including in utero exposure [48,49,50,51]. Fructose consumption acutely stimulates hepatic DNL in adolescents and adults [41,52,53]. In mice chronically consuming a high-fat diet (HFD), the addition of dietary sugars promoted triglyceride production [54,55]. Extra glucose promoted lipid synthesis through activation of the transcription factor carbohydrate-responsive element-binding protein (*Chrebp*), while extra fructose activated both *Chrebp* and sterol regulatory element-binding protein 1 (*Srebp1*) [54]. Dietary glucose and fructose exerted different effects on mitochondrial protein acetylation and malonyl CoA, resulting in a greater reduction in fatty acid oxidation and greater lipid synthesis in response to the extra fructose diet compared with the extra glucose diet [55].

It is perhaps not surprising that a reduction of dietary sugars results in several improvements in liver health. In one study, obese adolescents who were habitually high consumers of dietary sugar were placed on a prescribed diet for nine days that limited added sugar and fructose to 10% and 4% of energy intake, respectively [56,57]. In response to this short-term intervention, liver fat, DNL, and fasting insulin decreased, accompanied by a small (1.1 kg) amount of weight loss [56,57]. Subsequent interventions lasting eight weeks that were designed to be more sustainable in pediatric clinical settings yielded consistent results [58,59]. Schwimmer et al. [58] enrolled boys 11 to 16 years old with NAFLD and at least 10% hepatic fat content. They were randomly assigned to either a usual diet (control) or a low-sugar diet with a goal of less than 3% of daily energy from added sugars (intervention). For intervention participants, meals for their entire family were provided and existing foods with excess sugar were removed from their home. In response to the low-sugar diet, hepatic fat content was reduced by about 8% versus only 1% change in the control group. There was also greater reduction in plasma liver enzyme activities in the low-sugar group, but no significant changes were observed in fasting insulin or triglycerides. In a similar manner, Goss et al. [59] compared the effect of moderate reductions in dietary carbohydrate versus dietary fat in boys and girls with obesity and NAFLD. All meals were provided for the first two weeks and then families were given instructions on how to follow dietary guidelines for the remaining six weeks. Following the intervention, hepatic lipid was reduced in the low-carbohydrate group by 6% but only 1% in the low-fat group. The low-carbohydrate group also maintained fasting insulin sensitivity (HOMA-IR), whereas the low-fat group had an increase in HOMA-IR. Each of these studies demonstrate that dietary sugar reduction can lead to improvement in liver steatosis and potentially other health outcomes in obese adolescents.

Studies in humans have shown that individuals with NAFLD have low omega-3 polyunsaturated fatty acid (n-3 PUFA) intake and high n-6/n-3 PUFA intake ratio [60,61]. Owing to their effect on hepatic lipid metabolism and inflammation, n-3 PUFA as a nutrient supplementation has been recommended for improving NAFLD [62,63]. A randomized control study by Nobili et al. [64] reported that docosahexaenoic acid taken orally for 6 months reduced liver fat content and improved insulin sensitivity in children with NAFLD. Furthermore, Janczyk et al. [65] reported that although n-3 PUFA supplementation for 6 months did not improve steatosis as determined by ultrasound and ALT levels, it improved AST and gamma-glutamyl transpeptidase levels in children with NAFLD compared with placebo. Another study found that n-3 PUFA supplementation for 12 months had beneficial effects on steatosis and ALT levels in children with NAFLD and obesity [66]. Overall, nutrient intervention with modified PUFA levels appears to be safe and efficacious for the treatment of NAFLD in children. However, the real challenge for clinicians, behaviorists, and public health officials is to transfer these effects from small, highly controlled feeding studies to strategies that can be broadly implemented, with affordable, palatable, and sustainable diet options for families.

## 4. Mitochondrial Dysfunction and Oxidative Stress in the Progression of Pediatric NAFLD

Altered mitochondrial dynamics and function has been implicated in the pathophysiology of NAFLD, insulin resistance, T2D, and cardiovascular diseases in adults [67,68,69]. Examination of mitochondrial ultrastructure in NASH patients by electron microscopy identified the structural abnormalities characterized by the presence of megamitochondria with crystalline inclusions [70], swollen, rounded mitochondria, and loss of cristae [71]. Likewise, an in-depth investigation of mitochondrial ultrastructure in liver biopsy specimens from children aged 2–14 years with previously clinicopathologically-diagnosed NASH revealed numerous mitochondrial abnormalities such as adult NASH patients [72]. 

One emerging concept about NAFLD progression is that the liver makes several initial adaptations to nutrient overload and oxidative stress but eventually becomes overwhelmed and shows signs of decompensation. Mitochondria are intimately involved in this process. For example, Kolkaki et al. [73] performed detailed functional analyses of mitochondria acquired from liver samples from adults classified as normal-weight healthy controls, obese with or without liver steatosis, or obese with NASH. They found that mitochondrial respiration increased in the obese steatotic group compared with normal-weight and obese groups without steatosis. The NASH group had respiration rates similar to the normal-weight controls, despite ~30% higher mitochondrial mass, attributable in part to increased mitochondrial leak. Additionally, the NASH group had increased markers of oxidative stress, lipid peroxidation, and inflammation. These data support the concept that liver mitochondrial oxidative capacity in healthy people with obesity expands to accommodate increased carbon flux but eventually oxidative stress during NASH development causes mitochondrial failure. Further support for this idea was provided in a murine study, which demonstrated that elevated fatty acid availability caused the liver to increase its oxidative capacity, presumably to handle the excess lipid, but with the cost of increased oxidative stress and inflammation as carbon flux through anaplerotic reactions in mitochondria was increased [74]. The same authors reported that, in human liver samples, the calculated rate of oxidative flux was positively correlated with the severity of histopathology [74]. Additional human studies by the same group confirmed that oxidative flux through liver mitochondria was increased in people with liver steatosis compared with people without steatosis, and it was accompanied by increased anaplerosis, leading to increased gluconeogenesis and oxidative stress [38,75]. In rodents, the generation of mitochondria-derived damage-associated molecular patterns (mito-DAMPs) in the liver triggered inflammation and fibrosis [76]. That study also showed that mito-DAMPs were increased in humans with NASH and fibrosis. Thus, the liver responds to excess lipid or carbohydrate input through enhanced mitochondrial oxidation or anaplerotic carbon flux, but under chronic nutrient overload, oxidative stress leads to mitochondrial failure. In some animal studies, inherently low mitochondrial capacity predisposes the liver to oxidative stress, insulin resistance, steatosis, and fibrosis [77,78], but whether this is also the case in humans has not been demonstrated.

Oxidative stress plays a key role in the pathogenesis of NAFLD and progression to NASH (reviewed recently in [79]). Nobili et al. [80] found that oxidative stress is highly prevalent in pediatric NAFLD. Reductions in oxidative stress parameters have been observed in clinical trials in children with NAFLD after treatment with vitamin E plus hydroxytyrosol [81], calorie restriction with lycopene-rich tomato juice supplementation [82], and n-3 PUFA supplementation [83]. Furthermore, lipid oxidation products (LPOs) such as 4-hydroxynonenal and malondialdehyde are produced as a result of the interaction between reactive oxygen species (ROS) and PUFAs derived from membrane phospholipids [84]. The resulting LPOs generate mutagenic and carcinogenic exocyclic etheno-DNA adducts leading to hepatic carcinogenesis. LPO-mediated etheno-DNA adducts have been positively correlated with NAFLD in adults [85] and NASH in children [86]. Hence, oxidative stress along with ROS, LPOs, and etheno-DNA adducts are important mechanisms in the onset of NAFLD and progression to hepatocellular carcinoma.

## 5. Gut Microbial Dysbiosis in Pediatric NAFLD

Substantial evidence now indicates that an imbalance in the gut microbiota, i.e., microbial dysbiosis, plays a critical role in the development of NAFLD through altered energy homeostasis, exacerbated inflammation, and impaired choline and bile acid metabolism [87,88]; these alterations are beyond the scope of this review. In an analysis of the gut microbiome in children, NAFLD and its severity were associated with a greater abundance of genes encoding inflammatory bacterial products [89]. One pathway implicated in NAFLD pathogenesis is bacterial translocation, or the passage of intestinal bacteria into circulation [90,91,92]. In non-human primates (NHP), short-term fructose consumption increased serum levels of lipopolysaccharide (LPS) [93], an active component of the outer membrane of intestinal bacteria. Subsequently, elevated LPS promoted NASH independent of weight gain [93]. These relationships have been explored in humans as well. A handful of dietary analyses document inverse relations of healthy diets (e.g., Mediterranean diet) [94,95] and positive relations of sugar (fructose in particular [96,97]), fat [98,99], and total energy intake [100] with serum endotoxin. Higher serum endotoxin has been linked to NAFLD risk and progression in children [101,102,103] and adults [104,105,106,107]. Genes involved in LPS biosynthesis were enriched in the gut microbiome of children with NASH [89]. In rodents fed an HFD, translocation of gut microbial products into the blood may even precede obesity [108]. Short-chain fatty acids (SCFAs) butyrate, acetate, and propionate, produced by the fermentation of carbohydrates, can act in liver and adipose tissue to decrease expression of *PPARG* which in turn increases fatty acid oxidation [109]. However, children with NASH ferment dietary carbohydrates that augment oxidative stress through production of endogenous alcohol [110]. These SCFAs also have the potential to dampen immunity and reduce gut permeability [111], but their roles in pediatric NAFLD are complex and not well understood.

The maintenance of homeostasis and tolerance to environmental exposures controlled by immune mechanisms may be determined by microbial-host interactions occurring during a narrow time frame contained within the earliest days of life [112,113]. Studies in neonates have underscored the importance of microbial pioneers on chronic inflammatory disease development in later life [114,115]. Animal models suggest that gut microbiome dysbiosis in early life profoundly alters the development of the innate and adaptive immune systems [116]. Disruption of this early colonization process has been linked with childhood immunological diseases and contributes to an increased risk of metabolic diseases [117,118,119]. Lemas et al. [120] found that infants born to obese mothers have an altered gut microbiota including reduced Gammaproteobacteria, an aerobic LPS-producing microbe with an essential role in immune cell function. Germ-free mice colonized with stool from 2-week-old infants born to mothers with obesity showed elevated markers of inflammation and endoplasmic reticulum stress in their livers, and these mice progressed rapidly to NAFLD when challenged with a Western-style diet (WSD) [121]. Soderborg et al. also found that these colonized mice had increased periportal inflammation, which correlated with an increased modified pediatric NAFLD histological score [121] and increased collagen disposition around the portal triad [122] compared with mice colonized with stool from 2-week-old infants from normal-weight mothers. In the liver, LPS infiltration leads to pro-inflammatory cytokine production and liver Kupffer cell (KC) activation, which can lead to the activation of hepatic stellate cells and fibrosis [123]. These results support the concept that early maternal microbial dysbiosis contributes to the detrimental effects on offspring immune function that can drive hepatic inflammation. More research is needed, however, on the how changes in serum endotoxins, SCFAs, and microbial dysbiosis affect the mechanisms for pediatric NAFLD.

## 6. Micro-RNAs in Fatty Liver Disease: Predictors and Prognosticators

**Figure 1:**
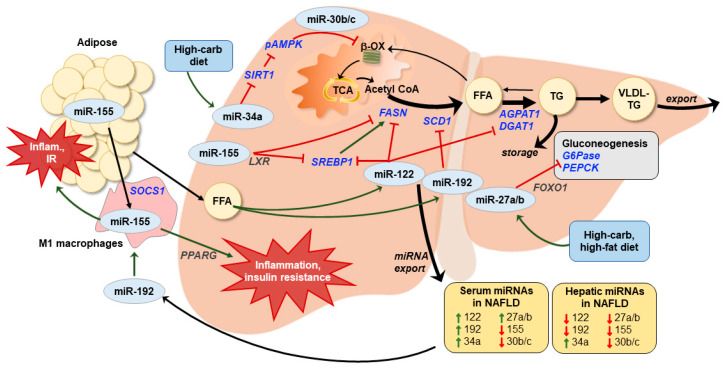
In normal liver, miR-122, -192, and -155 act as suppressors of lipid biosynthesis, while miR-27a/b suppresses gluconeogenesis. In the context of excess fatty acids and carbohydrates, as occurs during the development of non-alcoholic fatty liver disease (NAFLD)/non-alcoholic steatohepatitis (NASH), each of those miRNAs are found in reduced abundance within the liver, presumably due to excess export. The loss of the inhibitory actions allows for a metabolic shift in favor of lipid and glucose production and away from lipid oxidation. The increase in miR-34a, acting through the sirtuin 1 (SIRT1)/AMP-activated protein kinase (AMPK) axis, also contributes to a reduction in lipid oxidation. miR-155 and -192 play additional roles in activating M1 macrophages, contributing to the inflammatory and insulin-resistant milieu in adipose tissue and the liver. Green arrows reflect stimulatory actions and blunted red arrow represent inhibitory actions. β-OX, beta oxidation; FFA, free fatty acids; High-carb, high carbohydrate; Inflam, inflammation; IR, insulin resistance; miR, microRNA; miRNA, microRNA; TCA, tricarboxylic acid; TG, triglycerides; VLDL, very-low density lipoproteins.

MicroRNAs (miRNAs) are small (18–25 nucleotides) non-coding RNAs that regulate gene expression at the post-transcriptional level. The binding of miRNAs to specific messenger RNAs (mRNAs) inhibits translation, leading to reduced protein abundance of the target genes [124,125]. Each miRNA species has the potential to bind complementary sequences in multiple mRNAs and could influence several pathways. miRNAs can act both locally (intracellularly) and remotely through their secretion into the circulation and uptake into distant tissues. Blood-borne miRNAs are found within exosomes and bound to vesicle-free proteins and lipid particles, making them remarkably resistant to degradation [126,127]. The abundances of many miRNAs are altered in the liver and/or circulation in humans with NAFLD or animal/cell culture models of the disease, and several serum miRNAs have been examined as potential biomarkers [124,128,129,130]. We focus here on a few miRNAs with evidence to support their regulatory roles in the lipogenic, gluconeogenic, or β-oxidation pathways at the nexus of NAFLD development and progression (Figure 1). Importantly, most of the available evidence about the involvement of miRNAs in NAFLD comes from studies of adults or animals. We hypothesize that many of the same miRNAs operate similarly in pediatric NAFLD, but this remains to be determined.

miRNA-122 is the most extensively studied miRNA relevant to NAFLD and has the most promise as a biomarker because it is highly abundant in the liver and circulation [131,132]. It is produced almost exclusively by the liver, and its serum content increases with the presence and severity of NAFLD in adults [17,126,133] and animal models of NAFLD [127,134,135]. When considered separately or in combination with other biomarkers, miR-122 performed as well or better than ALT for predicting NAFLD severity [17,133]. The only published study of serum miR-122 content in pediatric NAFLD found that it was elevated in one cohort of children with the disease, but unaffected in another cohort, with no clear explanation for that discrepancy [136]. In adult human liver, miR-122 content is progressively reduced across the range from healthy to simple steatosis to NASH [127,137,138,139]. Initially, a healthy liver responds to rising levels of fatty acids by increasing miR-122 production, which then represses lipogenic enzymes and transcription factors such as *SREBP1*, fatty acid synthase (*FASN*), HMG-CoA reductase (*HMGCR*), and two enzymes required for triglyceride synthesis, 1-acyl-sn-glycerol-3-phosphase acyltransferase alpha 1 (*AGPAT1*) and diacylglycerol-acyltransferase 1 (*DGAT1*) [127,137]. This results in reduced hepatic lipid production and increased fat oxidation in normal liver [127,137]. However, longer-term nutrient overload hepatic miR-122 export accelerates and the loss of its inhibitory actions may contribute to exacerbated lipid production as NAFLD progresses to NASH and hepatic carcinoma [127,135]. The reason for excess miR-122 export in NAFLD is not yet established, although there is evidence that nutrient stress results in the upregulation of a protein that stabilizes miR-122 and directs it toward cellular export [140]. Strategies to maintain miR-122 abundance within the liver are currently being explored for hepatocellular carcinoma treatment [141,142].

miR-192 is also highly expressed in liver, has high serum content, and has promise as a biomarker for NAFLD in adults [17,126,133,143]. Since miR-192 is also abundantly produced in the gut, a change in serum miR-192 concentration may not always be the result of a change in liver health [132]. Nevertheless, serum miR-192 is increased in patients with NAFLD by up to six-fold compared with healthy comparison groups [17,126,133,143]. In animals and liver cell cultures, hepatocyte content of miR-192 declined in response to HFD or fatty acid exposure, resulting in the upregulation of one of its lipogenic targets, stearoyl-CoA desaturase (*SCD1*) [135,144,145]. Additionally, rats fed an HFD had increased liver miR-192 export, which activated M1 macrophages and resulted in increased liver inflammation [143]. In humans with NAFLD, serum miR-192 content was positively correlated with inflammation activity score and disease stage [143].

Other miRNAs are putative regulators of liver lipogenesis, gluconeogenesis, and fat oxidation. miR-155 represses *SREBP1* and *FASN* in hepatocytes by acting through the liver X receptor (LXR), leading to lower triglyceride production [146]. Adults with NAFLD had lower serum and liver miR-155 content than healthy controls, while liver transcripts for *SREBP1* and *FASN* were higher [146]. mIR-155 generation in adipose tissue is also involved in activating macrophages via the suppressor of cytokine signaling 1 (SOCS1) [129], leading to liver inflammation and insulin resistance in hepatocytes and adipocytes by targeting PPARG [129]. miR-30a promotes a shift from β-oxidation to DNL through the sirtuin 1 (SIRT1)/AMP-activated protein kinase (AMPK) pathway, and miR-30b/30c is an important co-regulator of that action [147,148]. miR-34a is increased in serum and liver from patients with NAFLD [137,149,150,151], while miR-30b/-30c is reduced in liver and serum [148,151]. miR-29a is involved in lipid oxidation through its action on the mitochondrial fat transporter, CD-36, leading to mitochondrial damage in mouse models [130]. In adults with NAFLD, serum miR-29a is increased and may be a biomarker for fibrosis and cirrhosis [130]. miR-27a and -27b regulate gluconeogenesis in mice by suppressing glucose-6-phosphatase (G6Pase) and phosphoenolpyruvate carboxykinase (PEPCK), two key enzymes responsible for hepatic gluconeogenesis [152]. This action is through forkhead box O1 (FOXO1). With chronic over nutrition, rodents display reduced liver content of miR-27a/27b while the serum level is increased [152,153]. Serum miR-27a/27b is also increased in adults with NAFLD or T2D, and children with obesity [133,152,154]. Lastly, miR-26a regulates multiple members of the hepatic gluconeogenic, β-oxidation, and insulin signaling pathways in mice [155]. In obese adults and obese mice, liver miR-26a content is reduced and restoring liver miR-26a rescues some of the adverse obesity-associated phenotype [155]. Recent work showed that the reduced liver miR-26a in obese mice also leads to an endoplasmic reticulum stress response, which can also be restored with miR-26a replacement [156]. Thus, an emerging picture is that oversupply of the liver results in increased content of several miRNAs, like those described above. This initial response helps suppress lipogenic and gluconeogenic pathways. However, with chronic overnutrition, several miRNAs are reduced in the liver, allowing the pathways they modulate to proceed unchecked. The loss of specific mRNAs in the liver is thought to be due to higher export rates to the circulation, which is why serum miRNA measurements have been tested as potential biomarkers of liver health in patients with NAFLD. 

Other lines of evidence support that miRNAs regulate metabolic function early in life. For example, during pregnancy, the fetus is exposed to miRNAs from the placenta and umbilical cord and the profile of several miRNAs in those tissues are altered in mothers with diabetes [157,158,159]. Hyperglycemia increased the placental expression of miR-130b, which in turn suppressed PPARG coactivator 1 alpha (*PPARGC1A*) [159], a master regulator of oxidative phenotype. As a result, the abundance of mitochondrial transcription factor A (*TFAM*) and mitochondrial DNA content was reduced in trophoblasts [159]. How this impacts the future health and development of offspring is yet to be determined. Another way in which infants are exposed to miRNAs is through mother’s milk [160,161]. miRNAs in milk are packaged in exosomes so they survive digestion in the infant gut [160]. A question to be resolved is whether the health of the mother alters the abundance of specific miRNAs in her milk and how that might affect the development of her offspring and impact their future risk for metabolic diseases.

## 7. Maternal Over-Nutrition and Developmental Programming of NAFLD

**Figure 2:**
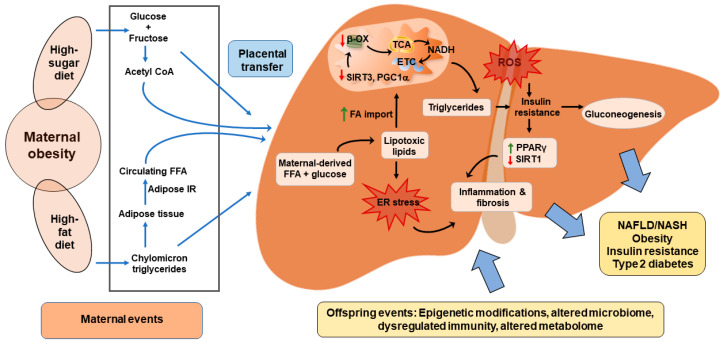
Maternal malnutrition imparts long-lasting detrimental effects on offspring liver. Placental transfer of excess circulating free fatty acids (FFA) and carbohydrates from obese mother induces mitochondrial dysfunction and endoplasmic reticulum (ER) stress. The resulting mitochondrial dysfunction predisposes the offspring liver to reduced lipid peroxidation by decreasing SIRT3 and PGC1α expression, oxidative stress, inflammation, insulin resistance (IR), and increased gluconeogenesis leading to the development of NAFLD. In utero exposure to an obesogenic environment also induces early microbial dysbiosis, leading to the reprogramming of offspring liver towards an inflammatory phenotype by epigenetic modifications. β-OX, beta oxidation; ETC, electron transport chain; ROS, reactive oxygen species; TCA, tricarboxylic acid.

Extensive animal and human evidence supports that maternal nutrition and obesity play key roles in the development of metabolic syndrome, insulin resistance, and NAFLD in offspring [11,162], summarized in Figure 2. Studies in humans demonstrated that increased intrahepatocellular lipid accumulation in neonates born to obese mothers correlated highly with maternal body mass index (BMI) [163,164]. Longitudinal, cross-sectional studies demonstrated that high or low birth weight is associated with greater odds of severe steatosis and fibrosis in adolescents with biopsy-confirmed NAFLD, independent of childhood BMI [165]. A variety of dietary approaches in animal models demonstrated a strong association between maternal obesity and the exacerbated risk of developing NAFLD in offspring [166,167,168,169]. Studies using NHP demonstrated that fetal offspring of HFD-fed dams show early signs of NAFLD; they had elevated liver triglycerides, markers of oxidative stress, and expression of gluconeogenic genes, independent of maternal obesity [170]. Interestingly, changing the maternal HFD to a low-fat diet during a subsequent pregnancy improved fetal hepatic triglyceride levels and gluconeogenic gene expression [170], suggesting that the maternal diet is a major risk factor for early NAFLD.

Experimental evidence from rodent models of maternal obesity strongly suggests impaired mitochondrial function as well as excess ROS generation in oocytes and zygotes [171] and in offspring at weaning [172] from diet-induced obese dams. Offspring exhibited peripheral insulin resistance as well as reduced liver mitochondrial DNA (mtDNA) and gene abundance of *Ppargc1a* [172], a key transcription factor in mitochondrial biogenesis [173]. Furthermore, oocytes and blastocytes from obese dams displayed reduced mtDNA, mitochondrial membrane potential, enhanced autophagy, and reduced mitochondrial biogenesis [174], which is linked to lipotoxic insult in liver. Interestingly, treating these obese mice with salubrinol, an endoplasmic reticulum (ER) stress inhibitor, before ovulation restored mtDNA content in association with enhanced mitochondrial replication factors, TFAM and DRP1 [174]. Likewise, Bruce et al. [168] observed that feeding HFD before or during pregnancy and lactation reduced hepatic mitochondrial electron transport chain activity and upregulated lipogenesis, driving the development of a NASH-like phenotype in offspring. In NHP, juvenile 1-year-old offspring from obese, insulin-resistant dams have a pattern of hepatic gene expression and cytokine and lipogenic responses, i.e., upregulation of *SREBP1*, *SREBP2*, *FASN*, *ACC1*, *LPIN1*, and *DGAT1* gene expression, consistent with the early stages of NAFLD, despite weaning to a normal diet at 7 months of age [175]. Furthermore, Alfaradhi et al. [176] demonstrated in mice that peripheral insulin resistance preceded by oxidative stress in offspring of obese dams triggers the expression of hepatic genes including *Pparg* to promote DNL in liver. In an elegant study, Ashino et al. [177] also observed the association between maternal insulin resistance and development of NAFLD in the mouse offspring of obese dams independent of post-weaning diet.

In addition to the miRNAs discussed above, NAFLD is associated with changes in DNA methylation in offspring from humans and animal models. One study in humans aimed at identifying transcriptional changes associated with offspring metabolic reprogramming identified changes in DNA methylation in genomic regions of liver transcription factors and monocyte differentiation [178]. Wankhade et al. [179] identified that a combination of maternal HFD and post-weaning methionine- and choline-deficient diet in mice altered the DNA methylation of liver transcription factors including *Ephb2* and *Vwf*, while maternal HFD alone changed the methylation of *Hnf4a*, *Ppargc1b*, and *Fgf21*. In mice with liver-specific IRS-1 deletion, parental insulin resistance was associated with epigenetically reprogrammed members of the TGF-β family, including neuronal regeneration-related protein (*Nrep*) and growth differentiation factor 15 (*Gdf15*), which modulate the expression of several genes involved in hepatic lipid metabolism [180]. In line with these data, in utero exposure to maternal HFD in NHP increased fetal liver *H3K14* acetylation concomitant with reduced *SIRT1* expression [181]. This reinforces the importance of parental nutrition/insulin resistance during pregnancy and lactation with epigenetic outcomes in offspring, but evidence in humans for a causal mechanism linking maternal obesity with offspring insulin resistance and pediatric NAFLD remains to be explored. 

## 8. Programmed/Trained Inflammation in the Pathogenesis of Pediatric NAFLD 

**Figure 3:**
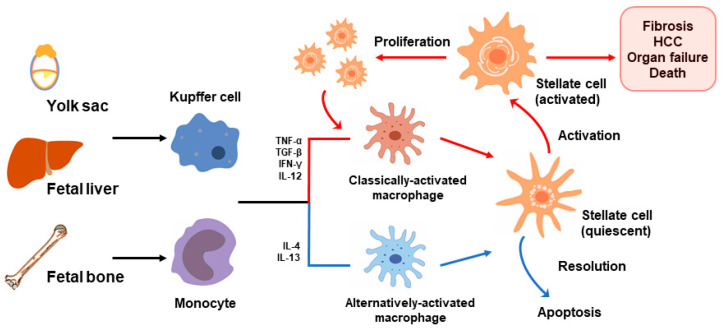
Bone-marrow-derived and liver resident macrophages in offspring from obese mothers have trained memories towards inflammatory phenotypes and secrete inflammatory and fibrogenic factors to induce hepatic stellate cell activation. Activated stellate cells undergo rapid proliferation to further worsen inflammation, ultimately leading to fibrosis, organ failure, and death. HCC, hepatocellular carcinoma.

A number of innate immune cells are involved in hepatic inflammation, particularly KCs, in children with NAFLD [182,183], summarized in Figure 3. In pediatric NASH, numerous activated macrophages are found in the spaces between damaged hepatocytes [184]. However, how portal inflammation develops in childhood NASH and differs from adult NASH is unknown. The liver harbors a large population of macrophages characterized by two distinct heterogeneous subsets: resident (KCs) and infiltrating macrophages [185]. Strong experimental evidence from both in vitro and in vivo studies indicates that KCs activate hepatic stellate cells to transdifferentiate into myofibroblasts, a major collagen-producing cell type in fibrosis, in an NF-κB-dependent manner in mice [186]. Stienstra et al. [187] identified that the depletion of KCs by clodronate liposomes in HFD-fed mice significantly reduced liver triglyceride levels with the concomitant upregulation of genes involved in fatty acid oxidation, including *Ppara* concomitant with the downregulation of *Il1b*. Mouralidarane et al. [169] showed that while exposure to maternal obesity followed by an obesogenic diet increased the number of KCs in mouse offspring liver, their phagocytic function was impaired and ROS production enhanced. Another study showed that the phagocytic function of KCs is significantly impaired in rats with diet-induced NASH and in patients with NAFLD regardless of KC numbers [188]. Impaired KC phagocytosis causes hyper-production of inflammatory mediators and activates hepatic inflammation leading to liver injury in NAFLD. In children with NASH, Lotowska et al. [184] found activated KCs in close proximity to transformed hepatic stellate cells and intensive fibrosis. 

Portal infiltration of macrophages is an early event in human NAFLD, occurring at the stage of steatosis before inflammation or fibrosis develops, and predicting progressive disease [189]. KCs and recruited circulating monocytes from the bone marrow (bone-marrow-derived macrophages (BMDMs)) both contribute to NAFLD pathology [190,191]. Studies in humans and in murine experimental models have shown that the hepatic recruitment of myeloid cells is regulated by chemokine receptor 2 (CCR2) and plays a critical role in obesity-induced inflammation by stimulating natural killer T cell apoptosis and releasing pro-inflammatory cytokines [192,193,194]. Recruited macrophages are highly plastic and acquire a distinct polarization state depending on the local microenvironment [195]. Mice exposed to maternal WSD have increased pro-inflammatory macrophage activation and in adulthood have increased recruited macrophages in the liver and accelerated hepatic fibrosis [196]. In another murine model, maternal HFD has been shown to restrict the expansion and renewal of fetal hematopoietic stem cells by altering the transcriptional output of genes regulating metabolism, stress response, proliferation, and other functions [197]. These data corroborate the premise that hepatic macrophages can be programmed by early diet exposure to retain a pro-inflammatory phenotype. Altogether, these data indicate the involvement of immune dysfunction in the pathogenesis of the development of NAFLD.

## 9. Conclusions

The pathophysiology of pediatric NAFLD is complex, multifactorial, and encompasses many unknown interactions between multiple cells types in the liver leading to NAFLD and inflammatory damage of hepatocytes. Cross-sectional studies have shown that the entire spectrum of NAFLD may occur during childhood, from hepatic steatosis to NASH to advanced fibrosis [198,199]. Despite advances in understanding adult liver pathophysiology, our understanding of pediatric NAFLD is incomplete for several reasons. First, most studies have focused on the endpoint of the disease and progressive factors have not been examined, leaving major gaps in our understanding of the many roles that make pediatric NAFLD unique from adult disease. Second, the majority of studies have examined processes in adult humans and animal models, and we know very little about critical physiological vulnerability during the perinatal period or critical transition periods such as puberty, which accelerate insulin resistance and disease patterns. Third, the lack of a suitable pediatric animal model is a major challenge for the field. Lastly, very few studies have been performed matching non-invasive discovery tools with cellular pathways for disease in individual pediatric NAFLD patients, one third of whom may advance very rapidly to NASH and cirrhosis. 

Maternal obesity is associated with increased risk for childhood obesity and NAFLD in both humans and animal models. Offspring from dams fed a WSD during pregnancy are programmed for inflammation, even if the offspring are switched to a normal diet at weaning [175,196]. However, the mechanisms by which maternal WSD exposure imparts lasting effects on the development and progression of liver disease in offspring are unknown. Both antenatal and postnatal factors have a profound impact on the developing infant gut microbiome and on immune cell development [200]. An understanding of how microbial-derived factors shape macrophage metabolism and polarization toward inflammation in the gut and other tissues is needed. Gut microbes and inflammation may play an active role in promoting the progression, if not the initiation, of NAFLD in children and adults, in part through epigenetic rewiring in liver and immune cells [201]. The release of macrophage-derived mediators contributes to inflammation and fibrogenesis, suggesting that cross-talk between macrophages, stellate cells, and endothelial cells plays a key role in the progression of NAFLD to NASH. DNA methylation, covalent modification of histones, and the expression of non-coding RNAs are the epigenetic phenomena that affect inflammatory processes in the context of NAFLD [202] and need to be understood further. Remodeling these pathways during pregnancy or lactation holds the promise for altered developmental programming in the next generation. Halting and reversing pediatric NAFLD/NASH will require identification of new biomarkers for individualized therapy. Understanding how miRNAs control metabolic and inflammatory pathways in liver and their cross-talk with other extracellular tissues, particularly in pediatric NAFLD, has received relatively little attention. Advancing our understanding of early-life contributors to pediatric NAFLD via maternal and childhood diet and obesity, the gut microbiota, and metabolic and inflammatory pathways is a critical and unmet need and should motivate future studies.
